# Mimicry of Food Intake: The Dynamic Interplay between Eating Companions

**DOI:** 10.1371/journal.pone.0031027

**Published:** 2012-02-01

**Authors:** Roel C. J. Hermans, Anna Lichtwarck-Aschoff, Kirsten E. Bevelander, C. Peter Herman, Junilla K. Larsen, Rutger C. M. E. Engels

**Affiliations:** 1 Department of Developmental Psychopathology, Behavioural Science Institute, Radboud University Nijmegen, Nijmegen, The Netherlands; 2 Department of Psychology, University of Toronto, Toronto, Ontario, Canada; University of Reading, United Kingdom

## Abstract

Numerous studies have shown that people adjust their intake directly to that of their eating companions; they eat more when others eat more, and less when others inhibit intake. A potential explanation for this modeling effect is that both eating companions' food intake becomes synchronized through processes of behavioral mimicry. No study, however, has tested whether behavioral mimicry can partially account for this modeling effect. To capture behavioral mimicry, real-time observations of dyads of young females having an evening meal were conducted. It was assessed whether mimicry depended on the time of the interaction and on the person who took the bite. A total of 70 young female dyads took part in the study, from which the total number of bites (N = 3,888) was used as unit of analyses. For each dyad, the total number of bites and the exact time at which each person took a bite were coded. Behavioral mimicry was operationalized as a bite taken within a fixed 5-second interval after the other person had taken a bite, whereas non-mimicked bites were defined as bites taken outside the 5-second interval. It was found that both women mimicked each other's eating behavior. They were more likely to take a bite of their meal in congruence with their eating companion rather than eating at their own pace. This behavioral mimicry was found to be more prominent at the beginning than at the end of the interaction. This study suggests that behavioral mimicry may partially account for social modeling of food intake.

## Introduction

A plethora of research has demonstrated that eating behavior is profoundly affected by social influences. Social facilitation research shows that the presence of others influences the amount of food eaten in a meal. Several studies have found that people eat more in the presence of others than when alone [1], [2]. Likewise, an individual's consumption can be modified by an eating companion; people tend to eat as much or as little as do those with whom they eat [3]–[5]. The process of adjusting one's intake to that of others is often referred to as modeling of food intake. These effects have been found to be robust and to override strong physiological influences [6]. Although the effects have been well documented, the underlying mechanisms are less clear.

Herman and Polivy [7] proposed a social-normative framework that assumes that people use other people's intake as a way of determining how much they may eat without appearing to eat excessively. What constitutes “appropriate eating” (and not excessive eating), however, is quite ambiguous and situationally dependent, so people often engage in social comparison. That is, they use the intake of others to determine what constitutes “appropriate eating” and adjust their own level of intake accordingly. This concern with eating appropriately is not misguided, and in particular not for women [8], because excessive eating often elicits negative stereotypes [9]. Although this normative framework provides a fairly simple, straightforward mechanism and explanation for modeling effects on eating, it is agnostic with respect to the dynamic processes that operate when two people are eating together. One possibility is that the intake of both eating companions becomes synchronized in real-time through behavioral mimicry. The principal aim of the current study is to test whether behavioral mimicry can (at least partially) account for modeling of food intake.

Behavioral mimicry refers to a process in which a person unwittingly imitates the behavior of another person. Research has shown that individuals automatically mimic many aspects of the people with whom they interact, including their postures, gestures, mannerisms, and speech accents [10]. This mimicry is assumed to occur because of the tight neural link between perception and action [11], [12]. That is, perceiving another person's movements activates one's own motor system for that same movement [13], which in turn increases the likelihood and ease of initiating a matched action [14]. In the domain of eating, seeing another person taking a bite might trigger a similar response in the perceiver, i.e. taking a bite as well. To the best of our knowledge, no studies in the field of social influences on food intake have tested whether people mimic the eating behavior of others in real-time (i.e., taking a bite when the other does). However, studies on alcohol consumption have investigated whether people mimic the drinking behavior of others. For example, Larsen and colleagues [15] examined whether young adults mimicked the sipping behavior of a same-sex peer during a 30-minute interaction. Their results showed that young adults were likely to take a sip directly after the other did. Koordeman and colleagues [16] demonstrated that young adults even mimicked the drinking behavior of movie actors while watching a one-hour movie, suggesting that mimicking the behavior of others can be triggered without a real-life interaction. These same perception-behavior linkages may operate in social eating contexts.

Although people often unwittingly imitate the behaviors of others, they do not mimic all the time [17]. Mimicry is increased in a situation in which there is a desire to affiliate with the interaction partner [10], [18]. Thus, when people have the motive to get along with their interaction partner, they are more likely to mimic that person. Next, it has also been found that individuals who were mimicked reported greater liking for those who mimicked them, and perceived their interaction with this person as having gone more smoothly [19]. These findings suggest that people may “use” mimicry to build liking and rapport with their interaction partner [18].

In order to capture behavioral mimicry processes in eating situations, real-time observations of dyadic meal interactions were conducted. There is ample evidence indicating that young adult females adjust their intake to that of their eating companions. This is the first study, however, that examines whether mimicry can (at least partially) account for these modeling effects. Based on the studies of imitation of alcohol consumption among young males and females [15], [16], we hypothesized that females would mimic the eating pattern of their eating companions by taking a bite after their eating companion had taken a bite. Moreover, to gain more insight into how situational factors might influence mimicry, we examined whether mimicry depended on the time of the interaction and on the person who took the bite. Because it is likely that winning the esteem of a previous-unknown interaction partner might be particularly evident at the beginning of an interaction, and it has been found that affiliation goals can augment behavioral mimicry [18], we hypothesized that young females would be more likely to mimic the bites of their eating companion at the beginning than at the end of the eating occasion.

## Methods

### Ethics Statement

The Ethical Committee of the Faculty of Social Sciences, Radboud University Nijmegen, approved the protocols for the present study. We obtained written consent of all participants involved in the study.

### Participants and design

The total sample consisted of 85 female dyads who were eating together during a 20-min eating occasion. This sample was part of an earlier study on the effects of portion size and the intake of others young women's food intake [20]. In this earlier study, naïve participants were paired with an instructed co-eater whose level of eating (i.e., small, medium or large amount) was determined by the experimenter. Further, the size of the initial portion was manipulated (i.e. small or medium-size portion). This eventually resulted in six different eating conditions. Because the co-eater did not receive instructions on when and how much bites she had to take from the meal, both women in the dyad can be seen as participants. Data from 15 dyads could not be used for subsequent analyses for the following reasons: (a) the videotaping equipment malfunctioned during the study (*n* = 10), (b) the DVD records were incomplete (n = 3), or (c) BMI values were missing (n = 2). The final sample, then, consisted of 70 same-sex dyads from which the total number of bites (N = 3888) was used. The mean age of each dyad was 21.62 (SD = 2.99).

### Setting and procedure

All sessions took place in the bar laboratory ( which is a replication of a real bar) at the campus of the Radboud University Nijmegen [21]. The bar was furnished with a table for two on which was placed a pitcher of water, two glasses, cutlery, two plates, a hot plate and some napkins. The chairs were situated facing each other so that both eating companions could easily see each other. Both women were served a complete meal; participants were free to eat as much or as little as they liked, whereas the overall intake of the instructed co-eater was determined by the experimenter. During each 20-min session, both women were observed by the experimenter from an adjacent room via a camera hidden in a lamp that was located next to the table. For each dyad, the experimenter coded the total number of bites and the exact time at which each woman took a bite.

### Measures

#### Timing and number of bites

First, we coded the exact time at which both women took a bite. A single bite was defined as a concrete touch of the fork to the mouth, while the food was cut with the teeth. Second, we counted the total number of bites taken by both women. To investigate behavioral mimicry, we distinguished between ‘mimicked bites’ and ‘non-mimicked bites’. Mimicry was operationalized as a bite taken within a fixed 5-second interval after the other person had taken a bite (also defined as the eating cue), whereas non-mimicked bites were defined as bites taken outside the 5-second interval. Previous studies on mimicry of sipping behavior have used 10- or 15-second time frames to answer comparable research questions [15], [16]. In the current study, however, a shorter time frame was used because bites during a normal eating situation appear to have a much higher pace than do alcohol sips. Therefore, to prevent overrepresentation of mimicry, a more stringent 5-second time frame was used.

#### Height and weight

In order to calculate both women's BMIs, the experimenter assessed height and weight following standard procedures [22]. Height was measured to the nearest 0.5 cm using a stadiometer (Seca 206, Seca GmbH & Co, Hamburg, Germany) and weight was measured to the nearest 0.1 kg using a digital scale (Seca Bella 840, Seca GmbH & Co, Hamburg, Germany). BMI was calculated as the weight in kilograms divided by the square of height in meters.

### Strategy for analyses

Because both women's bites were nested within the dyads, a multilevel framework was used for analysis. The dependent variable was dichotomous (i.e., mimicry versus no mimicry). The first aim was to test whether both women mimicked each other's intake. First, the total interaction time (i.e. 20 minutes) was divided into sensitive and non-sensitive periods. A sensitive period is a 5-second interval after one person within the dyad has taken a bite (sensitive in terms of the likelihood of mimicry), the non-sensitive periods are all of the remaining time periods after a bite. Thus, for each woman in the dyad we added all of the 5-second intervals (i.e. sensitive periods), this sum corresponds to the total number of bites the eating companion has taken. The non-sensitive periods are the remaining periods (i.e. total time in seconds ( = 1200) minus the sensitive periods). We then computed the ratio for the mimicked bites, which calculates how many bites a person has taken within those sensitive periods. A higher ratio means more mimicry. The ratio for the non-mimicked bites represents how many bites a person has taken in the non-sensitive periods (i.e. outside the 5-second interval after the eating companion has taken a bite). These two ratios were computed for both women separately. To examine whether both persons in the dyad were more likely to eat in the sensitive period than in the non-sensitive period, paired sample *t*-tests were computed comparing the ratios of the mimicked with the ratios of the non-mimicked bites. To examine whether both women in the dyad differed in the relative degree to which they mimicked the other person's bites, paired sample *t*-tests were computed comparing both women's overall bite ratios (i.e. mimicked bite ratio divided by non-mimicked bite ratio).

The second aim was to test whether the likelihood of behavioral mimicry depended on the time of the interaction and on the person who took the bite. To examine this question, the 20-min eating occasion was split into halves (i.e., the first ten minutes versus the second ten minutes). Further, each bite was assigned a 0 or 1 indicating who took the bite. A Multilevel Proportional Hazard Model (Cox regression) in a Survival Analysis framework was used to examine whether mimicry depended on the timing of the interaction (beginning or end of the interaction) and on the person who took the bite In contrast to the overall bite ratios, this analysis takes only the mimicked bites into account and therefore these results differ from the conducted *t*-tests. Data were analyzed using MPLUS 5.1 [23]. Because the physical appearance of the eating companion might have affected the extent to which individuals modeled the eating behavior of this person [24], [25], we controlled for both women's BMI scores in further analysis. Hazard ratios and Confidence Intervals were presented as effect sizes.

## Results

### Descriptives

On average, participants took 41.11 bites (SD = 13.34), whereas instructed co-eaters took an average of 30.13 bites (SD = 12.98) during the 20-minute eating occasion. This difference was significant, *t*_(69)_ = 6.53, *p*<.001). In terms of the total amount of food consumed, participants ate an average of 452. 13 grams (SD = 116.57) and instructed co-eaters 370.79 grams of food (SD = 211.27). The intra-class correlation showed that the amount eaten (in grams) by dyad members was significantly correlated, *r*_(70)_ = 0.52, *p*<0.001). It should be noted, however, that the instructed co-eaters' total amount consumed was determined by the experimenter. They were instructed to eat 125, 250, or 375 gram of food in the small-size portion conditions, whereas they were instructed to eat 250. 500, or 750 grams in the medium-size portion conditions. Across the eating occasion, significantly more bites were present in the beginning of the meal occasion compared to the end (3068 versus 820 respectively, *p*<.001). The difference over time in the number of bites does not affect the results of the survival analysis, because the likelihood of mimicry at a certain point in time is defined as the conditional probability of a mimicked bite given the number of bites during a particular time of the eating occasion.

### Do young women mimic the intake of their eating companion?

**Figure 1:**
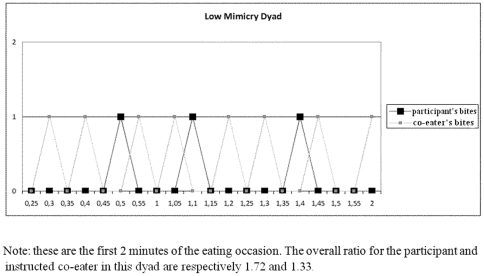
Example of behavioral data of a low-mimicry dyad.

**Figure 2:**
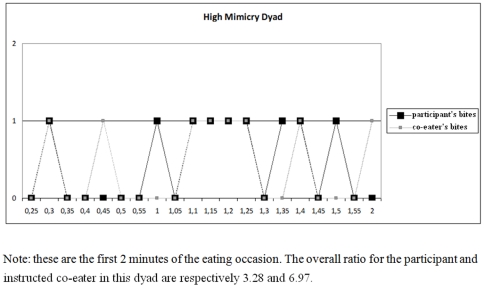
Example of behavioral data of a high-mimicry dyad.

The first aim was to test whether young women mimicked the intake of their eating companion. It was found that both women were significantly more likely to take a bite congruent with their eating companion's bite (i.e. within 5 s). (participant: *t*_(69)_ = 6.54, *p*<.001; co-eater: *t*_(69)_ = 8.67, *P*<.001). That is, they were more likely to take a bite when their eating companion was taking a bite rather than when the eating companion was not taking a bite. No differences were found between both women in the overall degree to which they mimicked their eating companion's bites, *t*_(69)_ = 1.81, p>.05). Figures 1 and 2 display examples of the behavioral data of high- and low-mimicry dyads.

### Does the timing of the interaction affect young females' likelihood of mimicry?

Second, we investigated whether the likelihood of behavioral mimicry depended on the time of the interaction and on the person who took the bite It appeared that women were more than three times as likely to mimic the intake of their eating companion at the beginning of the interaction compared to the end of the interaction (Hazard Ratio = 3.57, *P*<0.05, 95% CI = 2.23–5.72). The likelihood of mimicry was significantly higher when the instructed co-eater took a bite (Hazard Ratio = 1.93, *p*<0.001, 95% CI = 1.20–3.09). Further, a marginally significant interaction was found between the timing of the eating occasion and the person who took the bite (Hazard Ratio = 4.39, *P* = 0.054, 95% CI = 2.77–6.94). To further examine the interaction effect, we conducted separate analyses for the participants versus instructed co-eaters and first half versus second half of the interaction respectively. Throughout the interaction participants were significantly more likely to mimic the instructed co-eater than vice versa. Further, participants' as well as instructed co-eaters' likelihood of mimicry decreased significantly over time, whereas the decrease in mimicry was slightly more pronounced in the instructed co-eaters.

In additional analyses we also controlled for both women's BMIs. While controlling for BMIs, the effect of time remained significant (Hazard Ratio = 3.52, *p*<0.05, 95% CI = 2.2–5.63). Furthermore, it was still found that mimicry was significantly higher when the instructed co-eater took a bite of her meal (Hazard Ratio = 1.93, *p*<0.001, 95% CI = 1.20–3.09). Differences in BMIs also did not affect the interaction between the timing of the eating occasion and the person who took the bite (Hazard Ratio = 4.39, *p* = 0.06, 95% CI = 0.04–11.53). Thus, while controlling for differences in women's BMI, the results remained the same; mimicry was stronger in the beginning of the interaction and more likely to occur when the instructed co-eaters took the bite. Finally, because in the original study six different eating conditions were used, we also investigated whether the effects would be the same across conditions. The same model was run in all different eating conditions separately. The same pattern of results was found across conditions. The analyses for the separate conditions may be obtained from the corresponding author upon request.

## Discussion

Studies on modeling of food intake have consistently shown that young adult females eat more when their eating companions eat more and less when their eating companions eat less. The aim of the present study was to examine whether behavioral mimicry can (at least partially) account for these modeling effects of eating. Additionally, to gain more insight into how situational factors might influence mimicry, we examined whether mimicry of meal bites depended on the time of the interaction and on the person who took the bite.

First, the results showed that young females generally mimicked each other's eating behavior. That is, they were more likely to eat congruent (i.e. within 5 s) rather than incongruent with their eating companion. The matched actions of both eating companions fall within the typical definition of behavioral mimicry, i.e. the process in which a person unwittingly imitates the behavior of another person. Studies on human mimicry have explained this behavioral matching by proposing a mirroring network in which the perception of an action influences corresponding activation in the perceiver's motor system [11], [26], a process which is also known as the “perception-behavior expressway” [11]. The findings of the current study suggest that the same automatic perception-behavior linkages are also activated when two women are eating together. Thus, perceiving the eating companion taking a bite might have activated young women's motor system for the same movement, which in turn might have led to an increased likelihood of taking a bite as well. Another possibility is that that young women monitored each other's eating behavior in order to maintain a similar eating pattern. If the eating behavior of others communicates ‘appropriate’ eating, one's perceptions of another's behaviors might then be used to guide one's own eating behavior. This type of monitoring might fit into the normative framework of Herman and Polivy [7] that features individuals' desire to eat appropriately as an important determinant of their eating. Adjusting one's bites to that of others might be another solution (next to adjusting one's overall intake) to guard against overindulgence and to avoid the negative stereotypes that are associated with eating inappropriately [9]. It should be noted, however, that the current study did not test (or rule-out) whether young females' deliberately adjusted their behavior at such a micro-level or whether they unwittingly mimicked their eating companion's behavior.

Next, both women did not mimic the bites of their eating companion all the time. It appeared that both women were more than three times as likely to mimic the intake of their eating companion at the beginning of the interaction (i.e. first ten minutes) compared to the end of the interaction (i.e. last ten minutes). Previous studies have demonstrated that affiliation goals can augment behavioral mimicry [18], [27]. It is possible that young women's tendency to ingratiate themselves with their eating companion was especially marked at the beginning of the interaction, resulting in an increased likelihood of behavioral mimicry. By the same token, there might be less need to ingratiate at the end of the interaction, which might explain why the likelihood of mimicry diminished over the course of the interaction. The finding that this decrease was slightly more pronounced among the instructed co-eaters might be explained by the fact that the instructed co-eater was already acquainted with the study's procedure (i.e. eating with an unknown other), which in turn might have resulted in less prominent affiliation goals among the co-eaters. Although it is true that affiliation goals and rapport between two interaction partners are important moderators of mimicry effects, we would like to stress that this does not mean that mimicry requires rapport or affiliation goals to occur. We have articulated that the likelihood of mimicry diminished over the course of the interaction which might be due to the explanations given above. However, empirical studies are needed to gain more insight into why and under what circumstances people mimic each other's eating behavior. The potentially important role of conversation during the meal should be tested, for example in studies that investigate whether eating companions talk and eat in turns or might talk and eat in unison. These studies might examine the moderating effects of type of relationship (i.e. familiar or unfamiliar eating companions) and time spent on eating and talking on participants' synchronization of behavior.

Again, although the current study shows that behavioral mimicry may partially account for modeling of eating, we do not want to make the claim that all modeling effects on food intake can be explained by mimicry processes. Studies that simply made participants aware of how prior participants had behaved (‘remote-confederate design’) also found powerful modeling effects [28]–[30]. Insight into whether or not people are mimicking each other's intake, however, may help to resolve the question of whether large-eating companions allow their co-eaters to eat more or whether they force their co-eaters to eat more. Herman and colleagues [5] argued that, in the presence of palatable food, and in the absence of other constraints, people are motivated to eat as much as they want but that social norms serve an inhibitory function, indicating at what point one must stop eating in order to avoid excess. Thus, the large amount eaten by the eating companion allows people to eat more as well (without eating excessively). However, it is also possible that the large amount eaten by the eating companion does not simply allow to eat a lot, but virtually force one to eat a lot. Leone and colleagues [31] found that people who eat minimally are not particularly liked by their eating companions. Thus, if the other eats a lot, one might eat a lot as well (or at least not less than the other) in order to maintain a positive social relationship.

A few limitations warrant discussion. Although our findings suggest that affiliation goals might moderate mimicry of food intake, this was not specifically tested. To further understand the role of ingratiation attempts in explaining behavioral mimicry, future studies could specifically measure both eating companions' feelings toward each other and the quality of social interaction. This may give more insight into the possible bi-directional relationship between mimicry of food intake on the one hand, and affiliation goals or liking on the other. It would be interesting to compare those who mimicked with those who did not mimic in order to investigate the possible social bonding effects of mimicry in real-life eating situations. Second, the current study found no effect of weight status on people's tendency to mimic the eating behavior of their eating companion. It should be noted, however, that the research sample consisted of mostly normal-weight participants. Future studies are needed to examine whether normal-weight and overweight individuals differ in their likelihood of mimicry. In fact, it would be interesting to investigate whether similarities between both eating companions' physical appearance would influence behavioral mimicry effects. Third, the current study concentrated on young women. It is important to examine whether the same mimicry effects may be observed among other groups, such as children and adolescents. Because an important part of their socialization is acquired through the observation of their caregivers' and peers' behaviors [32], [33], and children and adolescents generally eat their meals and snacks in the presence of family members or peers at home or at school [34], [35], it is worth examining whether the same effects can be observed among these age groups. The current study used data from an experimental study in which young women were exposed to previously unknown eating companions. Although a highly natural, and thus generalizable, eating context was used, the question remains as to the extent to which family members, friends, or acquaintances would also mimic each other's eating behavior. In general, people should be more motivated to convey a good impression during their initial interactions with a stranger than with someone who they know well [36]. If behavioral mimicry reflects an attempt to ingratiate with others, we would expect less behavioral mimicry among familiar people than among strangers. Future studies, however, could examine whether this assumption is valid. Finally, one might argue that the specific eating context used in this study (i.e., dinner) facilitates behavioral mimicry. It would be interesting to replicate this study by using a different eating context in which, for example, individuals sometimes reach for palatable foods such as chips or sweets. If perceiving a nearby individual reaching for a snack results in a matched action, this might provide potential areas for interventions to prevent overconsumption of snack food.

All in all, our results suggest that behavioral mimicry may partially account for social modeling of food intake. Social modeling of food intake is a complex process, however, and may be explained from different theoretical perspectives. It seems to us that modeling can be both explained by norms regarding appropriate intake and social motives (affiliation/ingratiation) and that behavioral mimicry may underlie these processes, but that it depends on the context (i.e. whether or not the eating companion is actually present) which process (norms or social motives) is the most relevant. Nevertheless, insight into questions such as why people eat more or less just because someone else does or how mimicry develops over the course of an eating occasion has significant implications for one's health and well-being. The current study showed that people adjust their eating pattern to that of others. As long as such important influences on intake are not wholeheartedly acknowledged, it will be difficult to make healthy food choices and maintain a healthy diet, especially in eating contexts in which people are often exposed to the eating behavior of others.
